# Clinical Concept Extraction with Lexical Semantics to Support Automatic Annotation

**DOI:** 10.3390/ijerph182010564

**Published:** 2021-10-09

**Authors:** Asim Abbas, Muhammad Afzal, Jamil Hussain, Taqdir Ali, Hafiz Syed Muhammad Bilal, Sungyoung Lee, Seokhee Jeon

**Affiliations:** 1 Department of Computer Science and Engineering, Global Campus, Kyung Hee University, 1732 Deogyeong-daero, Giheung-gu, Yongin-si 17104, Korea; asimabbasturi@oslab.khu.ac.kr (A.A.); taqdir.ali@oslab.khu.ac.kr (T.A.); 2 Department of Software, Sejong University, Sejong 30019, Korea; mafzal@sejong.ac.kr; 3 Department of Data Science, Sejong University, Sejong 30019, Korea; jamil@sejong.ac.kr; 4 Department of Computing, SEECS, NUST University, Islamabad 44000, Pakistan; bilal.ali@seecs.edu.pk

**Keywords:** clinical concept extraction, data annotation, lexical semantics, medical concept classification, rule-based systems

## Abstract

Extracting clinical concepts, such as problems, diagnosis, and treatment, from unstructured clinical narrative documents enables data-driven approaches such as machine and deep learning to support advanced applications such as clinical decision-support systems, the assessment of disease progression, and the intelligent analysis of treatment efficacy. Various tools such as cTAKES, Sophia, MetaMap, and other rules-based approaches and algorithms have been used for automatic concept extraction. Recently, machine- and deep-learning approaches have been used to extract, classify, and accurately annotate terms and phrases. However, the requirement of an annotated dataset, which is labor-intensive, impedes the success of data-driven approaches. A rule-based mechanism could support the process of annotation, but existing rule-based approaches fail to adequately capture contextual, syntactic, and semantic patterns. This study intends to introduce a comprehensive rule-based system that automatically extracts clinical concepts from unstructured narratives with higher accuracy and transparency. The proposed system is a pipelined approach, capable of recognizing clinical concepts of three types, problem, treatment, and test, in the dataset collected from a published repository as a part of the I2b2 challenge 2010. The system’s performance is compared with that of three existing systems: Quick UMLS, BIO-CRF, and the Rules (i2b2) model. Compared to the baseline systems, the average F1-score of 72.94% was found to be 13% better than Quick UMLS, 3% better than BIO CRF, and 30.1% better than the Rules (i2b2) model. Individually, the system performance was noticeably higher for problem-related concepts, with an F1-score of 80.45%, followed by treatment-related concepts and test-related concepts, with F1-scores of 76.06% and 55.3%, respectively. The proposed methodology significantly improves the performance of concept extraction from unstructured clinical narratives by exploiting the linguistic and lexical semantic features. The approach can ease the automatic annotation process of clinical data, which ultimately improves the performance of supervised data-driven applications trained with these data.

## 1. Introduction

Entity and concept extraction from unstructured clinical documents are essential processes in a health informatics system [1]. The automatic extraction of an entity, concepts, and semantic relation from clinical documents enables a system designer to develop an accurate Clinical Decision-Support System (CDSS). Recently, numerous tools and algorithms such as QuickUMLS [1], Sophia [2], and cTAKES [3] have been broadly used in research and industrial applications to extract medical entities and concepts from unstructured clinical documents. Named entity recognition (NER) has received significant attention in the medical domain because it is a fundamental process for developing real-world applications such as CDSS. It is a complex task to identify an appropriate name for things, either conceptual or physical, from unstructured texts.

In the clinical domain, NER generally involves the extraction of concepts related to the “problem,” which consists of subclasses (signs or symptoms, findings, disease or syndrome, etc.), “treatment” (organic chemicals, diagnostic procedures, and/or pharmacological substances), and “test” (laboratory procedures and clinical attributes) [4]. These related concepts play a significant role in event detection, answering questions, information retrieval, and parsing tasks in the clinical domain. A knowledge-driven technique is extensively used to extract medical concepts, using existing numerous health and biomedical dictionaries and vocabularies such as the Unified Medical Language System (UMLS) Metathesaurus [5]. UMLS is a substantial medical knowledge source that consists of more than 6M names counted on 100 terminologies, more than 1.5M concepts, and 8M relations. A dictionary-based systems operation measures three aspects: (a) the size of vocabulary within a dictionary, (b) the matching algorithm, and (c) the scalability [6]. Many have focused on improving the precision and recall of information extraction systems, but less attention has been paid to the accuracy of their concept extraction.

In the clinical domain, everyday data are generated in an unstructured and heterogeneous format. According to a survey conducted between 2002 and 2015, most research has involved structured data rather than unstructured or clinical NLP data [7]. To acquire meaningful information from the clinical text, numerous rule- and lexical-based approaches have been practiced. Researchers have utilized rule-based methods, which have some limitations that require an expert to define rules and results in a new type of data. The set of rules usually numbers in the hundreds or thousands and is constructed by hand. We have defined dictionaries that can be shrunk concerning data requirements to overcome the limitations of rules-based data. Numerous clinical terminology and knowledge sources such as UMLS [5], MetaMap [8], and cTAKES [3] are extensively utilized to identify medical concepts. The selection of appropriate terminology or knowledge is also a challenge due to the high variability of clinical concepts. Extraction of knowledge from textual data and mapping them to some knowledge source is ongoing research in the biomedical and clinical domain that involves some NLP and text-mining techniques.

The primary aim of this work is to develop a system that automatically extracts and classifies clinical concepts, maintaining a high level of recall and precision, to contribute to the community of NLP and enhance current research in BioNLP. The second goal is to introduce a methodology representing standard concepts, semantic types, and entity types for medical phrases, by processing unstructured clinical documents.

This paper proposes a system that automatically identifies standard and meaningful clinical concepts from the UMLS Metathesaurus. We create rules that classify the extracted concepts into three categories: problem, treatment, and test. We use three existing clinical datasets to validate the proposed system: Beth Israel Deaconess Medical Center, i2b2 Test data, and Partners Healthcare (which consists of discharge summaries) [4]. 

The structure of the remainder of the paper is as follows: In Section 2, related works are discussed. In Section 3, the proposed methodology is presented. The experimental results and a discussion of existing and proposed methodology for clinical concept extraction and classification are given in Section 4. The conclusions and future work are discussed in Section 5.

## 2. Related Work

In the clinical domain, various NLP shared task challenges have been introduced for medical concept extraction, such as the i2b2 Challenge Shared Tasks [4] and ShARe/CLEF e-health Shared Task [9,10]. Previously, various traditional rule-based methods have been designed for NLP and text-mining research for unstructured clinical documents. Different tools such as MetaMap were introduced to identify clinical concepts from unstructured clinical documents that utilized UMLS terminology [11]. MetaMap 2013v2 experimented on the i2b2 2010 clinical dataset with the NLM 2013AB database and obtained low precision (47.3%) and recall (36%) scores.

Besides rule-based, machine-learning, ensemble-learning [12], and hybrid algorithms [13] have been introduced for concept extraction. However, we still identify some problems and limitations related to word-boundary identification and concept detection, and classification is needed to improve the precision and recall of the system. Other NLP tools and libraries such as Stanford parser [14], Lingpipe [15], c-TAKES [3], Splitter, Stanford CoreNLP, tree tagger, SPECIALIST, and open NLP are also used for text preprocessing and sentence- and word-boundary detection [16].

In the medical domain, various knowledge sources play a significant role in medical term matching and semantic-types mapping. UMLS is a versatile knowledge source, utilized for semantic-based concept mapping. The GENIA and i2b2 2010 datasets are extensively evaluated for unsupervised clinical NER [17]. Data heterogeneity in the clinical domain, clinical concept extraction, and classification are challenging and complex. In NLP research, common challenges and issues such as single or adjacent word-boundary identification have focused on assisting a CDSS system. 

In the following subsections, we discuss the strong and weak points of various clinical tools, approaches, and terminologies used for clinical concept extraction.

### 2.1. Information Extraction Clinical Tools

In the clinical domain, for clinical information extraction, the cTAKES [3], MetaMap [8], and MedLEE [18] tools are frequently utilized. cTAKES was developed by the Mayo Clinic but later became part of the Apache project. It was built on the UIMA (Apache Unstructured Information Management Architecture) framework and Apache OpenNLP toolkit open-source projects. It performs various linguistics and clinical tasks based on various analysis engines such as sentence tokenization, concept identification, NER, POS tagging, and normalization. cTAKES has played an essential role in the extraction of temporal relation discovery, patient smoking status, adverse drug events, and risk-factor identification utilizing EHRs data. However, the cTAKES installation process is complicated and needs additional effort to run.

The MetaMap tool was developed by the National Library of Medicine (NLM) and maps biomedical text exploiting UMLS services. MetaMap tool construction aims to enhance the biomedical-associated document retrieval operation from MEDLINE/PubMed. Subsequently, MetaMap was upgraded to deal with clinical text [19]. MetaMap has been employed in the literature review to support the emergency department, examine drug–disease treatment relationships, and aid with phenotype extraction and fragment identification by employing clinical documents, pharmacovigilance, and patient-associated characteristics extraction and it is known to be one of the primitive clinical NLP systems [20]. Kim et al. [21] used MetaMap 2013v2 to experiment on the i2b2 2010 clinical dataset with the NLM 2013AB database. It achieved low scores due to the concept and phrase-boundary definitions of MetaMap semantic categories not being thoroughly adjusted to the i2b2 concept definitions and being more sensitive to the lack of syntactic structure and the use of abbreviations [1].

Many other clinical tools such as OpenNLP [22] and NLTK [23] are available that focus on particular preprocessing tasks such as boundary detection, tokenization, and POS tagging. Our study utilized NLTK tools because the database is too straightforward for various NLP tasks. It is easy for users familiar with Python and can be a platform for developing research prototypes [24].

### 2.2. Clinical Information Classification and Extraction Methods

Recently, in the clinical domain, rule-based and machine-learning-based methods have been exploited to extract medical concepts and information.

#### 2.2.1. Rule-Based Approach

The main ingredient of a rule-based system is knowledge-based, relying on rules created by domain experts, and is considered highly efficient in exploiting language-related knowledge characteristics [25]. The previous research work has utilized the rule-based methods, which have some limitations like required knowledge expert to define rules and challenges like rule-based method effect results with a new type of data and time consumed building rule by hand which is often in hundred or thousand.

Recently, rule-based systems have been developed to identify peripheral arterial disease (PAD) by building regular expressions [26]. If the predesigned patterns match, PAD will be positively identified. Take the case of the diagnosis “severe atherosclerosis,” where “severe” results from a list of modifiers associated with positive signs of PAD, and “atherosclerosis” is from a personalized vocabulary constructed explicitly for the PAD task. Another rule-based system was introduced to expedite smoking status classification [27]. Early on, the researchers extracted the smoking status from each sentence and then identified the smoking status at the document level, employing precedence logic rules. The score of current smokers was significant, seek by a former smoker, nonsmoker and anonymous. For example, if a current smoker is extracted in a document from any sentence, the document will be labeled as a current smoker. The same logic rules are employed for the final patient smoking level status, i.e., classifying the patient as a current or former smoker. There are two approaches to constructing the rules, manual knowledge engineering or a hybrid system. A physician or expert must construct the rules in the manual knowledge engineering approach, which is time-consuming. A successful and highly accurate system can be designed by employing a knowledge engineering-based approach. Knowledge engineering stores and maintains a knowledge base in a structured database format such as UMLS [6]. The rule-based methodology in [28] has been introduced for three types of medical concept extraction: problem, treatment, and test. MetaMap, a medical terminology, has been utilized to extract the semantic features of a concept and then map it, employing rules. This methodology produces a very low precision score of 17%, with recall 18% for concept extraction. A rule-based methodology has been proposed for medical concept extraction from unstructured clinical notes utilizing UMLS. This methodology employs an exact match of the term to the UMLS to extract semantic information on the concept. Rules have been defined to map semantic information for concept classification. This methodology gains a precision score of 70% and an average recall of 60% [11], but the recall still needs to be improved to avoid missing information. A set of rules was constructed in [22] by extracting medical concepts from annotated training data. To extract and classify the medical concept, the author utilized a statistical technique. This technique yielded a minimal performance improvement, with accuracy, recall, and F1-Scores of 38.5%, 48.4%, and 42.9%, respectively. Instead of unstructured documents, the technique used structured annotated documents.

#### 2.2.2. Medical-Related Terminology

Medical terminology is a fundamental part of clinical text mining. In the healthcare domain, medical terminology is used to classify or extract information from clinical documents related to medication, treatment, disease, etc. There are many medical dictionaries available such as UMLS, LOINC, and SNOMED CT. The UMLS terminology thesaurus is mainly used in the literature in the clinical domain, for classification and extraction of information from clinical documents. A smartphone-based application has been developed that automatically extracts medical concepts, semantic-, and entity-type information from a medical text image utilizing UMLS. The medical text comprises a health report, a clinical case, and other kinds of medical-related texts. The limitation of this application is that it only extracts the concept and its semantic information using UMLS but does not classify concepts such as problem, treatment, or test [29].

The current version of UMLS contains more than 1 million concepts, 135 semantic types, and 54 relationships for concept categorization [30,31]. UMLS is a collection of distinct resource vocabularies such as Mesh, ICD-10, and SNOMED CT. UMLS is also employed to distribute and organize key terminology, coding standards, classification, and associated resources to construct more capable and interoperable biomedical information systems and services. 

An algorithm has been designed that automatically extracts medical concepts from unstructured clinical documents [32]. This algorithm presents a way to exploit UMLS to extract the standard concept and how its semantic information could be used efficiently in data-driven approaches. This algorithm merely classifies the concept into semantic and entity information in broad categories instead of a specific domain, or a clinical domain such as SOAP (Subjective, Objective, Assessment, Plan) or PICO (Problem, Intervention, Comparison, Outcomes). In the study by Campillos et al. [33], a harmonized methodology was introduced that automatically provides a semantic annotation to French clinical text utilizing UMLS. These tools produce semantic annotation data only for the French Language clinical corpus. Soldaini and Goharian [1] presents QuickUMLS tools for medical concept extraction, with an approximation term matching the UMLS method. They utilized a quantitative approach with a threshold value of 0.6–1.0 to choose an acceptable medical concept from a list of UMLS concepts. For huge-data scalability, QuickUMLS is the ideal option.

UMLS consists of three essential knowledge sources: Metathesaurus, Semantic Network, and Lexical Tools. UMLS provides a web browser, local installation, and UMLS Terminology Services (UTS) facility for a user to approach. The SPECIALIST Lexicon tool is employed to deal with NLP data. In the study by Liu et al. [34], a set of 163,666 abbreviations was extracted in more complete form pairs from UMLS.

## 3. Proposed Methodology

**Figure 1:**
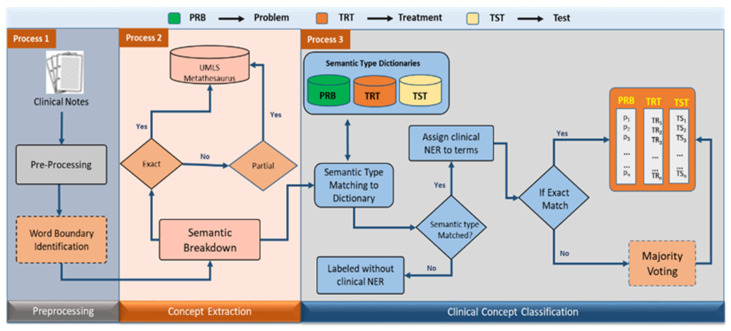
Proposed clinical concept extraction methodology: process 1 involves the preprocessing and word-boundary identification operation; process 2 performs semantic base concept extraction exploiting UMLS; process 3 involves clinical concept mapping.

We employed exact and approximate word matching to the UMLS Metathesaurus approach for semantic breakdown. The UMLS Metathesaurus is accessed using three variants: (a) directly, through a web browser, (b) by downloading the repositories for local use, or (c) by using the third-party web service API. Our study implemented the web service API, provided by the UMLS Terminology Service (UTS) [30] to access the concepts in the Metathesaurus. The proposed method consisted of three steps in a pipelined process involving preprocessing as the first step, concept extraction as the second step, and identifying the correct type of concept in the third step, as shown in Figure 1.

### 3.1. Document Preprocessing

A preprocessing operation such as tokenization, stop word removal, lemmatization, n-gram, or part-of-speech (POS) tagging is employed to improve the noisy data quality retrieved from unstructured narratives. Here, is a brief introduction to the notations used in the preprocessing step.

Let D = {d_1_, d_2_, d_3_ …… d_m_} denote the set of clinical documents, where dm denotes the mth clinical document, W = {w_1_, w_2_, w_3_ …… w_n_} denotes the set of words in a document, and w_n_ represents the nth word.

(a) Tokenization: Sentences are tokenized in each document into a set of words w_i_. All the stop words that convey no meaning, such as “the”, “this”, “from”, “on”, “off”, etc. are removed from the set W.
(b) Lemmatization: Words’ lemmas are identified to improve performance on ambiguous and invisible words. Lemmatization is preferred as it produces more accurate output compared to stemming in some instances, such as lemmatizing the word ‘caring’, it returns ‘care’, while stemming returns’ car’, and this is erroneous. We utilized the NLTK [23] WordNetLemmatizer package that provides a comprehensive and robust word-lemmatization solution.
(c) N-gramming: A word of n-gram is applied to represent a set of co-occurring words in a sentence, as described by Equation (1):
  (1) $$n=\;x\;\;\left(N\;–\;1\right)$$
  where **~** represents the subtraction of a scalar (*N* − 1) from each element of the vector $x\;=\;\sum_{k=0}^{n}\;W_{k}$.$\;W_{k}$ expresses the number of words in a sentence. We utilize four n-gram parameters because a medical concept can be a compound word such as “*overall left ventricular systolic function*”.
(d) Deduplication: Duplicate words are removed to reduce the data dimensionality and avoid ambiguity. We utilized a set of built-in data type functions with characteristics to store data in an unordered and unchangeable way that would not allow duplicate values.
(e) POS tagging: The part of speech (POS) tagging using NLTK NLP library was employed and then we constructed a regular expression pattern to filter only meaningful information such as nouns, adjectives, and adverbs from a list of words, as shown in Equation (2). <NN*> denotes all the noun phrases, “<JJ*>” represents all the adjectives, and “<RB*> shows the adverb phrases from X, where X represents the “bag of words” list attained through regular expression.
  X = Bag of words = “< NN∗ >< JJ∗ >< RB∗ >”(2)

#### Word Boundary Detection

**Figure 2:**
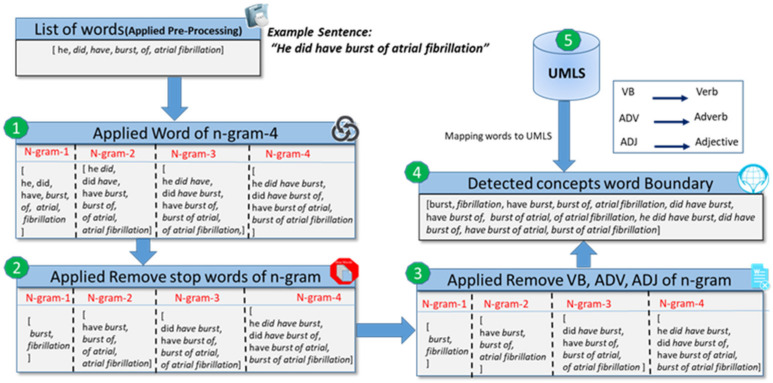
Concept word-boundary identification methodology:identify the word boundary applying to the preprocessing; and map each word to the UMLS.

In the clinical domain, word boundary detection is a process of detecting single or multiple adjacent words that indicate a clinical concept. Multiple adjacent words can be a mixture of stop words, punctuations, and digits representing a clinical concept, making it challenging in the information extraction domain. We developed a procedure, illustrated in Figure 2, to smoothly identify the boundary of a single or multiple adjacent words of a clinical concept by employing rules and regular expressions.

(a) Preprocessing: A preProcessing procedure is created that accepts an unstructured clinical document as an input ingredient to preprocess. Subsequently, this is applied to preprocessing steps such as tokenization, lemmatization, etc. We obtained a bag of words with a size of n-gram-4, as described in Section 3.1.
(b) Stop-words removal: In the preprocessing step in Section 3.1, we did not apply the stop-word removal operation because multiple adjacent word concepts also contained stop words such as *“a pelvic fracture,”* where *“a”* is a stop word. In the second step, we removed stop words of n-gram from the list of n-gram words such as “is the”, “did have of”, etc.
(c) Stop words and POS filtering: A word that appears with a combination of stop word, verb, adjective, and adverb that does not convey a domain knowledge discarded such as “of atrial”, “good effect”, or “very good effect”, as described in Algorithm 1, step 9.
(d) Detected words boundary: We retained a list of alternate words that contains either stop words or not, such as “have burst”, “burst of atrial”, etc., because these words convey a domain of knowledge related to a heart problem. In another method, we identified noun phrases and eliminated all other phrases.
(e) Word mapping to UMLS: Finally, we mapped each word to the UMLS to extract semantic information, practicing exact and approximate word matching.

**Algorithm 1**: Clinical concepts—word-boundary identification.   **Input**: Unstructured Clinical Document2.    **Output**: Word boundary identification1.  wordList ← new ArrayList< >2.  wordSet ← new ArrayList< >3.  Doc: Read Document4.  bagOfWords ← preProcessing(Doc)5.  **for each** word in bagOfWords, **do**6.    **if** word Not Equal to Null, **do**7.    **for each** word_2 **in** word.split(), **do**8.      w_tag ← **pos_tag**(word_2)9.     **if** word_2 in (stopWords) OR w_tag == (Verb, Adjective, Adverb), **do**10.      wordSet ← word_211.      **end if**12.       **end for**13.      **if** len(wordSet) Not Equal to len(word.split()), **do**14.       wordList ← word15.      wordSet.clear()16.      **end if**17.    **end if**18. **end for**


### 3.2. Clinical Concept Extraction

The semantic breakdown is achieved by utilizing the UMLS Metathesaurus for concept categorization and standard concept extraction. Clinical concept extraction is a multistep process that includes finding terms, concept identification, semantic-type extraction, and entity-type extraction.

#### 3.2.1. Finding Terms

Each word in *X_i_* (bag of words) is mapped to the UMLS Metathesaurus. As described in Equation (3), if a match is found in the UMLS, a sequence of terms is stored in term list T; otherwise, *X_i_* is omitted from the list.

(3) $$X_{i}\;\in UMLS\;?\;T\;\leftarrow \;X_{i}:pop\left(X_{i}\right)$$

#### 3.2.2. Concept Identification

In the UMLS Metathesaurus, a concept demonstrates the meaning of medical terms by various names. The importance of the Metathesaurus is to illustrate the predefined meaning of each name and associate all the names from the entire source vocabularies that provide similar meaning, called synonyms. Each concept in the Metathesaurus occupied a permanent and unique concept identifier represented as “name,”, e.g., *“Coronary Arteriosclerosis”*. When a new concept is added into the Metathesaurus structure, each concept is attached with a unique identifier (“ui”) value such as “*C0010054*”. In the Metathesaurus, there is a single concept or a list of concepts available for each term, as shown in Box 1.

Box 1 **Entity-type extraction from UMLS Metathesaurus.**

“Input” ⟶ Coronary artery disease        (Term)   [

{

“ui”: “ C0010054 ”,                      (Concept ID)

"rootSource": "MTH",

"uri":"https://utsws.nlm.nih.gov/rest/content/2019AB/CUI/ C0010054 ",

"name": "Coronary Arteriosclerosis"        (Concept Name)   },

]

A stepwise process of concept identification from UMLS is presented in Algorithm 2. The input ingredients of Algorithm 2 are a set of clinical documents represented by *D*, and the algorithm output is a set of terms and concepts. We read the documents (Doc), subsequently applied to preprocess, and Algorithm 1 produces a list of words that will be obtained and stored in a *wordList* array. A loop is applied to read each word as a *term* from a *wordlist* array. For each term, we identified a correspondent concept ID and a concept name from UMLS and stored it into *cui*, and a *concept* variable for the given *term* is set as a parameter in the *searchConceptUMLS method*. If the length of cui is not zero, we applied a check, mapped the *term*, *cui*, and *concept* into *conceptMap* and *cuiList* array, and read the next term. If the length of *cui* was null, the next term should be read. The loop was continued until the entire *wordList* was read.
**Algorithm 2**: Concept Identification from UMLS.  ***Input****: Clinical Document D* ← {d_1_, d_2_, d_3_.... d_n_} *# set of documents d_i_*   ***Output****: set of Terms and Concepts**1.*  *wordlist* ← *newArrayList<>**2.*  *conceptMap* ← *newMultiMap<term, concept >**3.*  Doc: Read Document*4.*  *wordList* ← *Pre-Processing(Doc)**5.*  **for each** *term*
***in*** *wordList*, **do***6.*   **String**: *cui, concept* ← ***searchConceptUMLS*** (parameter: *term*)*7.*   **If** *size(cui)* > **0**, **do***8.*     *conceptMap<k, v>* ← *term, concept**9.*    *cuiList* ← *cui**10.*    **Next***term**11.*    **end if***12.*  **end for**

#### 3.2.3. Semantic-Type Identification

Semantic type plays a crucial role in concept categorization, such as medical problems, medical treatment, and medical tests [15], as it gives an interpreted and obvious meaning to Metathesaurus concepts [30,35]. For instance, for the general term “Trout”, the semantic type is “fish”, but not “animal”—why? Because “fish” conveys a closer meaning to the concept “trout” than “animal”. Each concept has at least one semantic type (STY) in the Metathesaurus and a maximum of five semantic types [30,36]. A multifarious or inherently vague concept consists of more than one STY, such as “Febrile Convulsion,” which is a concept of “Finding” as well as “Disease or Syndrome” [35]. In the example shown in Box 2, semantic types acquired from UMLS are based on the “Concept ID” or “*ui*” for the term “Trout”.

Box 2 **Semantic-type extraction from the UMLS Metathesaurus.**

“Input": ⟶ Trout          (Term)

{

"classType": "Concept",

“ui”: “C0041200”,           (Concept ID)

“semanticTypes”:  [  {

“name”: “Fish”,       (Semantic Types)

“uri”: “https://uts-ws.nlm.nih.gov/rest/semantic-network/2019AB/TUI/T013”      (Semantic Type ID)

          }     ],

“name”: “Salmo trutta”        (Concept Name)

}

The semantic-type extraction process for each clinical concept is presented in Algorithm 3, where the input information is a list of concepts IDs extracted through Algorithm 2 and the output is a set of semantic types corresponding to each concept. Each concept ID is interpreted as cui from a *cuiList*, used afterward in the searchSemanticTypeUMLS method to retrieve the concept name and the semantic type. Concept names and semantic types are stored in the data-collection arrays, represented as *semanticTypeMap* and *SemanticTypeList*. The next cui is read until the entire *cuiList* is processed.
**Algorithm 3**: Semantic-Type Identification from UMLS. ***Input:***
*List of Concepts ID’s C* ← {c_1_, c_2_, c_3_ … c_n_}   ***Output****: set of Concepts ID’s (cui) and Semantic types**1.*  *semanticTypeMap* ← *new MultiMap<conceptID, sematnicType >**2.*  *semanticTypleList* ← *new ArrayList<>**3.*  *cuiList:* Read cuiList from Algorithm.2 *4.*  **for each**
*cui*
***in*** *cuiList*, **do***5.*   **String**: *conceptName, semanticType* ← ***searchSemanticTypeUMLS*** (parameter*: cui*)*6.*     *semanticTypeMap<k, v>* ← *conceptName, semanticType**7.*    *semanticTypeList* ← *semanticType**8.*   **Next***:cui**9.* **end**

#### 3.2.4. Entity-Type Identification

An entity type demonstrates the parent relation for a concept. In contrast to the semantic type, entity types represent the meaning of the concepts in a more standard, explicit, and precise form [30]. In a Metathesaurus, each concept has only one entity type. In the example shown in Box 3, using semantic type ID “*ui*”, the entity type is extracted from the Metathesaurus and presented as “*expandedForm*” along with other information such as “*definition*” and “*abbreviation*”.

Box 3 **Entity-type extraction from UMLS Metathesaurus.**

{“Input": ⟶ Fish         (Semantic Type)     {

"ui": "T013",            (Semantic Type ID)

"definition": "*A cold-blooded aquatic vertebrate characterized by fins and breathing by gills. Included here are fishes having either a bony skeleton, such as a perch, or a cartilaginous skeleton, such as a shark, or those lacking a jaw, such as lamprey or hagfish*",

"semanticTypeGroup": {

"abbreviation": "LIVB",

"expandedForm": "Living Beings",       (Entity Type)

},

"name": "Fish"    }}

Algorithm 4 is designed to describe the entity-type identification process from UMLS. The input ingredients for the algorithm are a list of semantic types S (the output of Algorithm 3), and the output is a set of entity types for corresponding semantic types. A loop is applied to read each semantic type from a *semanticTypeList* array and extract the entity type as *entityType* from UMLS for the corresponding semantic type. The *semanticType* and *entityType* are mapped into the *entityTypeMap* array list. The loop continues until the entire *semanticTypeList* is finished reading.
**Algorithm 4**: Entity-Type Identification from UMLS. ***Input****: List of Semantic Types S* ← {s_1_, s_2_, s_3_ … s_n_}   ***Output****: set of Semantic Types (STY’s) and Entity types**1.*  *entityTypeMap* ← *new MultiMap<semanticType, entityType >**2.*  *semanticTypeList:* Read semanticTypeList from Algorithm.3*3.*  **for each**
*type*
***in*** *semanticTypeList*, **do**
*4.*   **String**: *sematnicType, entityType* ← ***searchEntityTypeUMLS***  (parameter*: type*)*5.*   *entityTypeMap<k, v>* ← *semanticType, entityType**6.*  **Next***:type**7.* **end**

#### 3.2.5. Example Case Study

**Figure 3:**
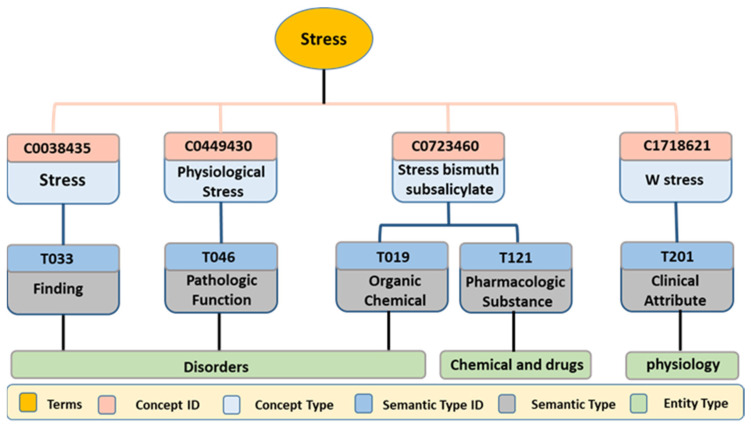
UMLS Metathesaurus concept-extraction case study hierarchical tree.

An example case study is demonstrated in hierarchal tree form, as shown in Figure 3, for enabling the proposed algorithms to extract standard forms of the terms from UMLS Metathesaurus. The medical term “*Stress*” is taken as an example to demonstrate the process of identification and extraction. First, the term “*Stress*” is submitted to the UMLS Metathesaurus to identify the related concepts. The Metathesaurus acknowledges a list of concept IDs as “*ui*” and concept names as “*name*”, along with other information such as “*root resource*” and “*uri*”. As shown in Figure 3, the term “*Stress*” was divided into four concepts (names and identifiers) in the UMLS Metathesaurus. The concept ID’s Metathesaurus caters for semantic types and gives a set of helpful information for each concept. Each concept has only one semantic type, while the concept “*Stress bismuth subsalicylate*” consists of two semantic types “*organic chemical*” and “*pharmacological substance*”. The semantic type IDs associated with each semantic type are utilized to find the entity type, which delivers more standard and interpreted context for a medical concept.

### 3.3. Clinical Concept Classification

Concept extraction and classification have been adopted to extract and classify clinical information from a text for a wide range of applications, ranging from supporting clinical decision making to improving the quality of care. A rule has been constructed to map the semantic information of medical phrases to semantic dictionaries, as shown in Table 1.

A set of semantic-type mapping rules is constructed for medical terms to classify them into explicit categories. The mapping dictionaries are enriched with semantic type for three domains: problem, treatment, and testing. 

Three dictionaries, as shown in Table 1, i.e., PRB ← Problem Dictionary, TRT ← Treatment Dictionary, and TST ← Test Dictionary, are searched for semantic types.

**Figure 4:**
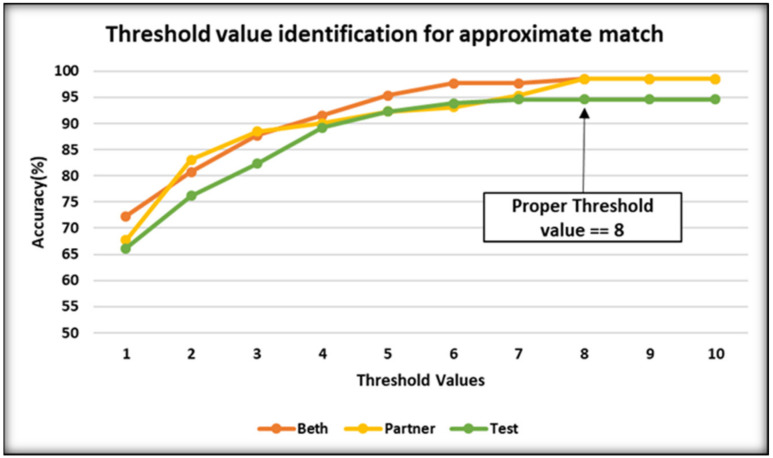
Appropriate threshold-value identification employs an approximate term matching approach to UMLS.

While searching for a term, several concepts are returned from the Metathesaurus, and approximate string matching is employed to reach the final decision. The first eight concepts, along with their semantic type, were selected and labeled in the retrieved concept list. We selected the first eight concepts because the Metathesaurus reflects and preserves the meanings, concept names, and relationships from its source vocabularies. It does not represent a comprehensive NLM-authored ontology of biomedicine or a single, consistent view of the world. It stores all the meanings and content of its source vocabulary in a single common format. The native format of each vocabulary is carefully studied and then inverted into the common Metathesaurus format. For some vocabulary, this involves representing implied information in a more explicit format. For example, if a source vocabulary stores its preferred concept name as the first occurrence in a list of alternative concept names, that first name is explicitly tagged as the preferred name for that source. Eight is chosen as a threshold value after the evaluation on three datasets with concepts retrieved from UMLS, as shown in Figure 4, where the accuracy reaches the peak value and remains stable afterward. We evaluated gold datasets provided by the i2b2 National Center, Partners Healthcare, and Beth Israel Deaconess Medical Center [4] to identify the threshold value for concept retrieval. A precision and recall protocol was used to measure the accuracy against each threshold value in the range between 1 and 10 in the x-axis and the level of accuracy is shown on the y-axis in Figure 4. Subsequently, in semantic labeling, these concepts are categorized into a Problem, Treatment, or Test, using the rules described in Table 2, Table 3 and Table 4. In case of an overlapping situation where a concept could be mapped to more than one category, we implemented a majority voting technique. Equation (4) shows the concept identification with the highest frequency for each category.

For instance, the concept “stress” maps to [“problem”, “problem”, “test”, “treatment”]. The argmax function assigns “problem” to *T*.

(4) $$T\;\leftarrow argmax[\overset{N}{\underset{i=1}{\sum }}Ci]$$

Suppose we have classes: c1 = “Problem”, c2 = “Treatment”, c3 = “Test” and T = “Term”. Then:

**Rule 1:**
  If the number of any concept type (**c1**, **c2**, **c3**) is greater for the term T, then classify the term with the majority-threshold concept.
  For instance, if **c1 > c2 AND c1 > c3** then **T ← c1**, else if **c2 > c1 AND c2 > c3** then **T** **← c2** else if **c3 > c1 AND c3 > c2** then **T** **← c3**.

**Rule 2:**
  If the number of any concept type (**c1**, **c2**, **c3**) is similar for term T, then presume a first-class (c1) as a majority threshold.
  Such as: IF frequency of **c1 == c2 == c3 then T** **← c1 OR c2 OR c3,** which means that the class that is on the first index in a list will be selected as a perfect class for a concept.

**Rule 3:**
  If the number of two concept types (**c1**, **c2**) is similar, we ignored the third concept type (c3) and assigned the first concept type(c1) to the term between two similar concept types (c1, c2).
  **Such as**: IF number of **c1 == c2 AND c3 < c1 AND c2**, ignore **c3** and assigned **T← c1 OR c2**, else if number of **c2 == c3 AND c1 < c2 AND c3**, ignore **c1** and assigned **T← c2 OR c3**, else if number of **c1 == c3 AND c2 < c1 AND c3**, ignore **c2** and assigned **T← c1 OR c3**.

**Figure 5:**
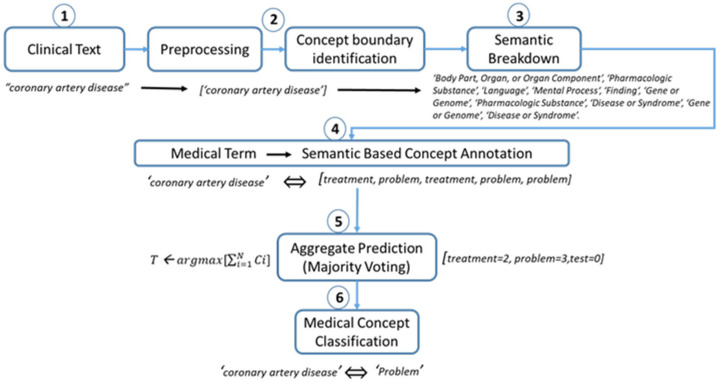
A step-by-step process of medical concept classification using majority voting.

Figure 5 demonstrates the stepwise process of classification for the clinical term “coronary artery disease”. After preprocessing and boundary identification, a list of entity types was obtained through a semantic breakdown process. Each entity type was mapped to a corresponding clinical domain (problem, treatment, and test) using the PRB, TRT, and TST dictionaries, as discussed in Section 3.3. The number of entries was counted in each domain, and through the majority voting technique, the final category was chosen. As described in Figure 5, the term “coronary artery disease” was classified into the “problem” domain because its maximum score (argmax: 3) was greater than that of the other two domains.

## 4. Results and Discussion

The empirical analysis of the proposed system methodology involved evaluating unstructured clinical documents provided by the i2b2 National Center in 2010 NLP challenges. We utilized the NLTK library for NLP and the text-mining process in the Python programming language environment; the source code is available on GitHub for research purposes (https://github.com/TuriAsim/Medical-Concept-Extraction-and-Classification) (accessed 1 September 2021).

### 4.1. Performance Measures

To measure and compare system performance, generally, three indexes are used for information retrieval and extraction: precision, recall, and F1-score. Precision measures the number of valid instances in the set of all retrieved instances. Recall measures the number of valid instances in the intended class of instances. F1-score is the harmonic mean between precision and recall, with β = 1 used to obtain the adjusted F-score. The measures can be computed through the following equations for a balanced dataset:

(5) $$F1-\mathrm{score}=2*\frac{\mathrm{Precision}*\mathrm{Recall}}{\mathrm{Precision}+\mathrm{Recall}}$$

(6) $$\mathrm{Precision}=\frac{\mathrm{TP}}{\mathrm{TP}+\mathrm{FP}}$$

(7) $$\mathrm{Recall}=\frac{\mathrm{TP}}{\mathrm{TP}+\mathrm{FN}}$$

### 4.2. Datasets

We evaluated three unstructured clinical datasets provided by i2b2 National Center, Partners Healthcare, and Beth Israel Deaconess Medical Center to measure the system performance. The clinical dataset consisted of discharge summaries that were annotated manually for three types of clinical concepts (problem, treatment, test) according to the instructions of the i2b2/VA challenge organizers [4]. Partners Healthcare contains 97 annotated notes, Beth Israel Deaconess Medical Center contains 73 annotated notes, and the test dataset provided by i2b2 National center for system evaluation contains 256 annotated notes. We utilized both training and test annotated notes for experimental purposes, and the gold dataset was used for the evaluation as shown in Table 5.

### 4.3. Word-Boundary Identification Algorithm Performance

To calculate the efficiency and accuracy of the boundary identification algorithm in measuring index sensitivity, we utilized a gold dataset provided by i2b2. We selected 20 unstructured documents from each of the three test datasets (Beth medical center, Partner healthcare, and i2b2 test dataset) and processed them through the proposed algorithm. The average performance of the algorithm on the datasets, in terms of the sensitivity score, was 97.14% as shown in Table 6.

(8) $$\mathrm{Sensitivity}=\frac{Number\;of\;true\;positives}{\left(Number\;of\;true\;positive+Number\;of\;false\;negatives\right)}$$

### 4.4. Semantic Breaking

**Figure 6:**
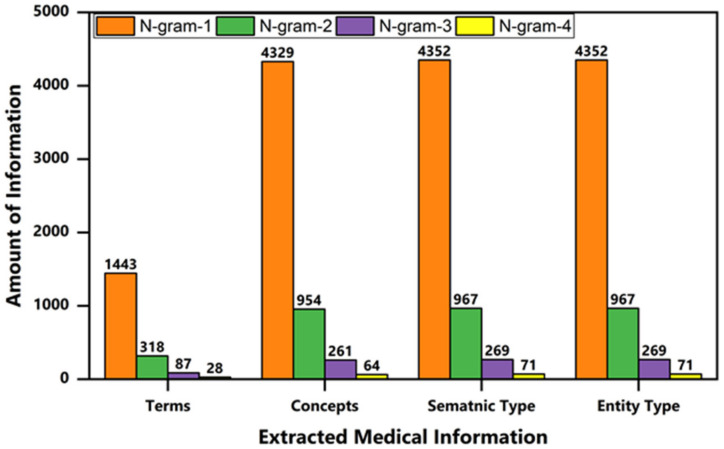
UMLS semantic breakdown using n-gramming strategies with n = 1–4.

We performed the semantic breakdown analysis using n-gram combinations. We picked up 10 unstructured clinical notes from the i2b2 dataset for experimental purposes after preprocessing a bag of words obtained in the range of n-gram-4. Each word was mapped to UMLS, and information was extracted such as concepts, semantic types, and entity types. In a subsequent analysis, we found that the UMLS Metathesaurus identifies medical terms using n-gram-1 quantitatively better than n-gram-2, n-gram-3, and n-gram-4. However, the terms with n-gram > 1 deliver extra meaningful and coherent information for the user. For example, “blood pressure,” “coronary artery disease,” and “liver function test normal” are more meaningful terms as compared to a single term such as “pressure,” “blood,” “coronary,”, etc. As the n-gram word size increases, the matching accuracy of composite terms to the Metathesaurus decreases, as shown in Figure 6.

#### 4.4.1. System Performance Comparison with Competitors

We compared our proposed system’s performance with that of three related systems: QuickUMLS [1], BIO-CRF [28], and the Rules (i2b2) model [22]. The three systems were tested against the i2b2 2010 dataset for three types of concept category extraction (problem, treatment, and test). 

Quick UMLS employed an approximate dictionary matching approach for medical concept extraction. It required a threshold value between 0.6 to 1.0 to select an acceptable medical concept from a collection of UMLS concepts. We used both approximate dictionary matching and exact word matching in the suggested methodology, which resulted in 25% greater accuracy and 12% higher recall when compared to Quick UMLS. In addition, Quick UMLS had 4% greater recall compared to the proposed methodology. 

**Figure 7:**
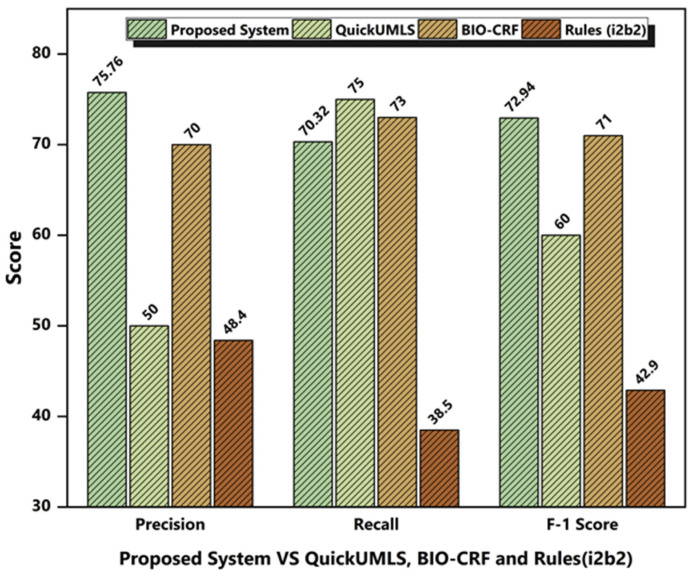
Performance comparison of the proposed system with the QuickUMLS, BIO-CRF, and Rules (i2b2) models.

The rules (i2b2) model created a simple set of rules by harvesting information from the annotated training data. This rule-based algorithm used a statistical method to categorize and extract concepts from structured and annotated data. Our suggested rules-based methodology used a majority vote mechanism to identify and extract concepts from unstructured clinical data. When compared to the rules (i2b2) model, the proposed methodology yielded higher precision, recall, and F1-score, as shown in Figure 7. 

BIO-CRF is a medical concept extraction approach based on machine learning. It aims to automatically identify the concept boundary and assign the concept type to them. 

For each medical concept, word-level and orthographic-level features were retrieved to train the BIO-CRF model. At the individual concept and dataset level, we compared the performance of the proposed approach with BIO-CRF. The proposed methodology achieved 75.76% precision and a 72.94% F1-score, which are approximately 6% and 2% higher than the BIO-CRF system, respectively, while BIO-CRF achieved approximately 4% higher recall than the proposed system. Overall, the proposed system performed better than the QuickUMLS, BIO-CRF, and Rules (i2b2) models, as shown in Figure 7.

#### 4.4.2. Performance Comparison on Individual Concept Extraction

We measured the proposed system performance on individual concept extraction and compared it with that of the runner-up system, BIO-CRF [4]. As described in Table 7, the precision score was similar for the proposed and BIO-CRF systems in the “problem” category. However, the proposed system showed better performance in terms of increased recall of 14% and an F1-score 7% higher. Measuring the performance for “treatment” concepts, we found that the BIO-CRF performance was better, with 11% higher precision than the proposed system, but the proposed system outperformed the counterpart in terms of recall with a 17% increase and with an F1-score 2% higher.

The proposed system and BIO-CRF produced similar precision results of 80%, but BIO-CRF performed better in terms of recall, with a 25% increase, and the F1-score increased by 18% for individual concepts in the “test” category.

#### 4.4.3. Independent System Performance

We also calculated the results of individual systems for three functions: exact match, approximate match, and exact-plus-approximate match, and calculated the average precision, recall, and F-score. For this experiment, we used an imbalanced dataset. Imbalance means that the number of concepts available for three categories (problem, treatment, and test) is different in a single clinical note. For example, if the number of problems is 30, treatments is 20, and tests is 15, we can say that the dataset is imbalanced.

(9) $$\;\;Average\;Precision\;f\left(P\right)=\frac{1}{N}\overset{N}{\underset{i=1}{\sum }}\frac{TPi}{TIi}\;\;$$

(10) $$Average\;Recall\;f\left(R\right)=\frac{1}{N}\overset{N}{\underset{i=1}{\sum }}\frac{TPi}{TTi}$$

(11) $$\;\;\;\;Average\;F-\mathrm{Score}\;f\left(\mathrm{fscore}\right)=2*\frac{f\left(P\right)*f\left(R\right)}{f(P+f\left(R\right)}$$

where *N* represents the number of classes, TI is the total number of inferred labels, and TT is the ground truth label.

Subsequently analyzing the results of exact and approximate term matches to UMLS individually, we discovered that much information was missing when employing exact term matching to UMLS with a precision of 87.66%, recall of 44.62%, and F1-score of 59.14%. Later, we added approximate term matching to the UMLS approach. We acquired a lower precision (71.53%) then with exact matching but had an improved recall of 63.53% and F1-score of 67.29% then exact term matching. Therefore, we concluded that precision and recall needed to be improved. We employed a hybrid methodology that merged the exact and approximate term-matching mechanisms to UMLS. After preprocessing, word boundary detection was employed to extract a list of terms obtained. Each term was matched to the UMLS by two approaches, an exact match and an approximate match. When the exact match approach was used, matched terms were listed. Again, the approximate match approach was used on the other unmatched terms. In the end, these two lists were combined from the exact and approximate matching of terms, and rules were applied as discussed in Section 3.3.

**Figure 8:**
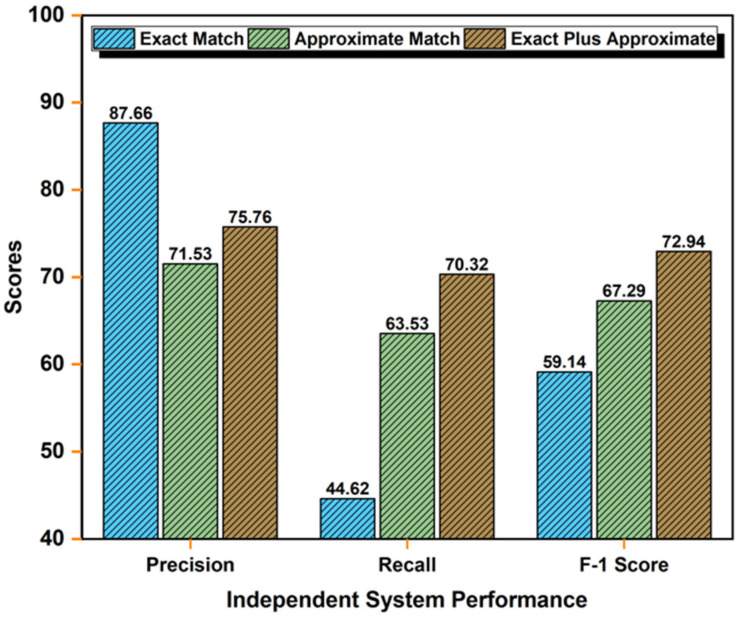
Individual system performance for exact, approximate, and exact-plus-approximate term matching to UMLS mechanism.

We found that the hybrid methodology produced a dramatic increase in results, with high precision of 75.75%, recall of 70.32%, and F1-score of 72.94%, as shown in Figure 8.

The individual datasets and concept wise results is attached in appendix for further deep analysis and discussion as shown in Appendix A.1, Appendix A.2 and Appendix A.3.

## 5. Conclusions

We proposed an extensive rule-based system that automatically identifies clinical concept word boundaries and extracts and classifies clinical concepts by exploiting UMLS. We measured the system performance in various ways, such as individual concept-wise, dataset-wise, and system-wise, and compared the proposed system results with other existing systems and methodologies in the same domain, such as QuickUMLS, BIO-CRF, and the Rules (i2b2) model. The overall proposed system performed better than the QuickUMLS, BIO-CRF, and Rules (i2b2) models, achieving high precision of 76.76%, recall of 70.32%, and F1-score of 72.94% for individual concepts. We also constructed results for concept word-boundary identification and achieved a sensitivity score of 97.14%. Rules are generalized instead of domain-dependent regardless of the semantics of the statement. The proposed system utilized UMLS to identify and extract standard and semantic information. This study can play an essential role in automatically extracting, classifying, and annotating or labeling medical data for data-driven approaches such as deep learning and machine learning. We did not employ a machine-learning algorithm; therefore, the performance is documented and size-independent.

Although the proposed system’s reliability and accuracy have been proven by this research outcome, there were also some limitations to the research. A strict word boundary and composite word detection were challenges for a well-defined concept such as “saphenous vein graft -> posterior descending artery.” These errors can be resolved in the future by employing some regular expression patterns and deep-learning algorithms such as word embedding. Due to the homogeneous structure of the clinical documents and the huge dataset, the system processing time was increased. Some computational preprocessing needs to be performed to clean the data and convert them into a structured database format. The system processing time and efficiency will be boosted and measured in the future to expedite the processing of massive datasets.

## Figures and Tables

**Table 1:** Medical concept semantic-type dictionaries for concept classification.

Clinical Domain	Semantic Type
Problem	“Disease or Syndrome, Sign or Symptom, Finding, Pathologic Function, Mental or Behavioral Dysfunction, Injury or Poisoning, Cell or Molecular Dysfunction, Congenital Abnormality, Acquired Abnormality, Neoplastic Process, Anatomic Abnormality, virus/bacterium.”
Treatment	“Therapeutic or Preventive Procedure, Organic Chemical, Pharmacologic Substance, Biomedical and Dental material, Antibiotic, Clinical Drug, Steroid, Drug Delivery Device, Medical Device.”
Test	“Tissue, Cell, Laboratory or Test Result, Laboratory Procedure, diagnostic procedure, Clinical Attribute, Body Substance.”

**Table 2:** The implementation scenario of Rule 1.

Process Name	Processes
Clinical Term	beta blockers
Semantic Breakdown	beta blockers = [‘Pharmacologic Substance’, ‘Organic Chemical’, ‘Pathologic Function’, ‘Organic Chemical’, ‘Clinical Attribute’, ‘Injury or Poisoning’, ‘Pharmacologic Substance’]
Semantic-Based Concept Annotation	beta blockers = [‘Treatment’, ‘Treatment’, ‘Problem’, ‘Treatment’, ‘Test’, ‘Problem’, ‘Treatment’]
Majority Voting	beta blockers=argmax (‘Treatment’ = 4, ‘Problem’ = 2, ‘Test’ = 1)
Classification	beta blockers ⇔ Treatment

**Table 3:** The implementation scenario of Rule 2.

Process Name	Processes
Clinical Term	heart rate
Semantic Breakdown	heart rate = [‘Clinical Attribute’, ‘Clinical Attribute’, ‘Finding’, ‘Finding’, ‘Medical Device’, ‘Medical Device’]
Semantic-Based Concept Annotation	heart rate = [‘Test,’ Test’, ‘Problem’, ‘Problem’, ‘Treatment’, “Treatment’]
Majority Voting	heart rate=argmax (‘Test’ = 2, ‘Problem’ = 2, ‘Treatment’ = 2)
Classification	heart rate⇔ Test

**Table 4:** Present the implementation scenario of Rule 3.

Process Name	Processes
Clinical Term	increased heart rate
Semantic Breakdown	increased heart rate = [“Finding’, ‘Finding’, ‘Clinical Attribute’, ‘Clinical Attribute’, ‘Finding’, ‘Clinical Attribute’, ‘Finding’, ‘Clinical Attribute’, ‘NONE’, ‘NONE’]
Semantic-Based Concept Annotation	increased heart rate = [‘Problem’, ‘Problem’, ‘Test’, ‘Test’, Problem’, ‘Test’, ‘Problem’, ‘Test’]
Majority Voting	increased heart rate=argmax (‘Problem’ = 4, ‘Test’ = 4, ‘Treatment’ = 0)
Classification	Increased heart rate ⇔ Problem

**Table 5:** All the datasets and their details, provided by i2b2 National Center.

Data Source	Golden Datasets	Number of Concepts			
		Problem	Treatment	Test	Total
Beth Medical Center	73	4187	3073	3036	10,296
Partners Healthcare	97	2886	1771	1572	6229
i2b2 Test dataset	256	12,592	9343	9226	31161
Total	426	19,665	14,187	13,834	47,686

**Table 6:** Overall datasets and individual performance on the i2b2 datasets for word-boundary identification algorithm.

Datasets	True Positive	False Negative	Sensitivity
Beth Medical Center	255	7	97.33%
Partners Healthcare	162	5	97%
i2b2 Test Dataset	400	13	96.85%
Overall Results	817	24	97.14%

**Table 7:** Individual concepts’ performance measurements: proposed system vs. BIO-CRF model.

Systems	Category	Precision (%)	Recall (%)	F1-Score (%)
BIO-CRF	Problem	79%	69%	74%
	Treatment	79%	70%	74%
	Test	80%	67%	73%
Proposed System	Problem	79%	83%	81%
	Treatment	68%	87%	76%
	Test	80%	42%	55%

**Figure A1:**
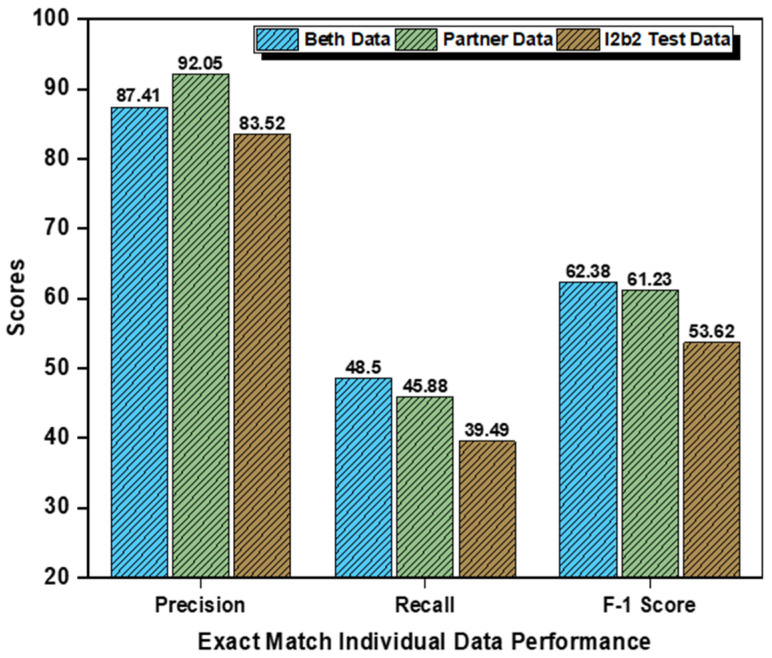
Individual dataset performances, employing exact term matching to UMLS.

**Figure A2:**
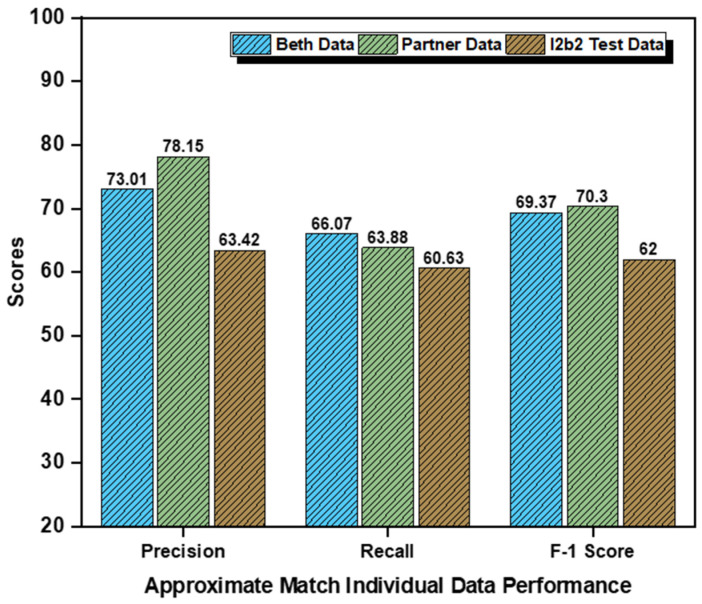
Individual dataset performances, employing approximate term matching to UMLS.

**Figure A3:**
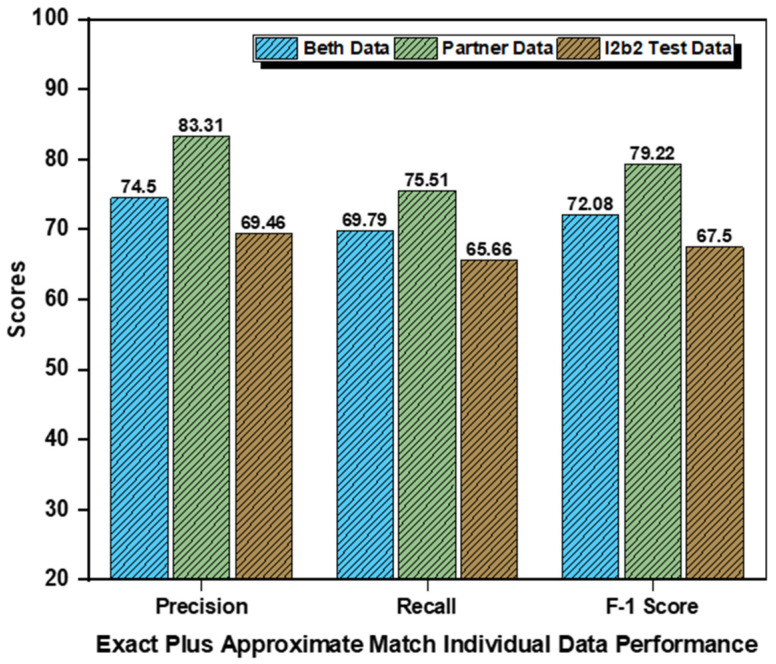
Individual dataset performances, employing exact and approximate term matching to UMLS.

## Data Availability

We have utilized the i2b2 2010 shared challenge a well known dataset available on (https://www.i2b2.org/NLP/DataSets/ (accessed 1 September 2021)).

## Appendix A

### Appendix A.1. Performance on Individual Datasets Using Exact Matching

We also evaluated the proposed system on different datasets, employed an exact term matching approach, and achieved an overall recall below 50% for all datasets, which means that more than 50% of concepts missed and did not match UMLS. Employing an exact matching mechanism is obligatory to see improvement. The proposed system had a higher precision of 90.05% for Partner datasets than the Beth and test datasets, while it achieved a higher F1-score of 62.38% for Beth compared to Partner and Test data (see Figure A1).

We also computed the individual concept-wise performance for each dataset by employing the exact word-matching approach shown in Table A1. Comparable precision of 94% was produced for problems evaluating the Beth, Partner, and Test datasets. Less than 50% recall was measured for the Problem concept in all three types of datasets, but we calculated a higher F1-score of 65% in the Beth dataset for the Problem concept. High precision of 93.91% was determined for the Treatment concept in Partner data. An almost equal recall of 56% was found in the Beth, Partner, and Test data, with a high F1-score of 70.46% noted in Partner data for the individual concept treatment. A comparable precision of 89% was noted for the Beth and Partner data, while the overall recall was found to be below 50% for the three types of dataset for the Test concept, and a high F1-score of 56.19% was calculated for the Beth data for the Test concept.

**Table A1:** Individual concepts’ performance measures for the Beth, Partner, and Test datasets, employing exact term matching to UMLS.

Datasets	Categories	Precision (%)	Recall (%)	F1-Score (%)
Beth Medical Center	Problem	94.83%	49.55%	65.09%
	Treatment	78.13%	54.95%	64.52%
	Test	89.27%	40.99%	56.19%
Partners Healthcare	Problem	93.33%	47.93%	63.34%
	Treatment	93.91%	56.37%	70.46%
	Test	88.89%	33.33%	48.48%
i2b2 Test Data set	Problem	93.33%	36.27%	54.24%
	Treatment	71.80%	56.57%	63.28%
	Test	85.41%	25.63%	39.43%

### Appendix A.2. Individual Datasets and Concept-Wise Performance Approximate Matching

As discussed in Section 4.4.3, the recall for concept extraction needs to be improved by exploiting approximate term matching to UMLS. Several concepts have been missed due to imprecise composite word-boundary identifications or concepts not being precisely matched to UMLS when employing exact term matching.

An approximate term matching using UMLS for medical concept matching was employed to improve recall and precision. We analyzed the results of the proposed system for approximate matching and performed experiments for individual datasets and concepts. We achieved a precision of 78.15% and recall of 70.3% for Partner data, which was higher than for Beth and Test data, but obtained a 66.07% recall for Beth data, which was higher than for Partner and Test data, as shown in Figure A2. We also measured the individual concept-wise performance for all three types of datasets. Overall, the Partner dataset obtained the highest precision of 84.46% and 75%, recall of 86.23% and 94.29%, and F1-score of 85.34% and 83.55% for an individual concept’s problem and treatment, which was better than the performance of the Beth and i2b2 Test datasets. Beth data achieved the highest precision of 85%, recall of 39.08%, and F1-score of 53.54% for the individual concept test, being overall better than the Partner and Test data for every concept (see Table A2). The overall i2b2 test dataset returned low scores for precision, recall, and F1-score for all the individual concepts in problem, treatment, and test.

**Table A2:** Individual concepts’ performance measures for the Beth, Partner, and Test datasets, employing approximate term matching to UMLS.

Datasets	Categories	Precision (%)	Recall (%)	F1-Score (5)
Beth Medical Center	Problem	62.73%	83.47%	71.63%
	Treatment	71.31%	75.65%	73.42%
	Test	85%	39.08%	53.54%
Partners Healthcare	Problem	84.46%	86.23%	85.34%
	Treatment	75%	94.29%	83.55%
	Test	75%	11.11%	19.35%
i2b2 Test Dataset	Problem	67.05%	78.28%	72.23%
	Treatment	50.81%	77.04%	61.23%
	Test	72.41%	26.58%	38.89%

### Appendix A.3. Individual Datasets and Concept-Wise Performance: Exact-Plus-Approximate Matching

We computed the results, individual dataset-wise and concept-wise, utilizing the Beth, Partner, and Test datasets. As a result, high precision of 83.31%, recall of 75.51%, and F1-score of 79.22% were measured for the Partner dataset, which was better than for the Beth and Test data when combining exact and approximate term-matching approaches in UMLS. An overall low score was calculated for the Test dataset with a precision of 69.46%, recall 65.66%, and F1-score of 67.5%, as shown in Figure A3. We also computed results for individual datasets concept-wise, followed by exact-plus-approximate concept matching in UMLS. We performed the analysis in each dataset for the individual concept, noting higher precision of 91.67% and 78.26%, recall of 86.52% and 100%, and F1-scores of 89.02% and 87.80% for problem and treatment in the Partner dataset compared with the Beth and Test dataset. Individual concept Test gained a higher precision of 85.42% in Test datasets and achieved a high recall of 44.26% in the Beth dataset. An equal F1-score of 56% was calculated for the Test concept in the Beth dataset and the Test data, as shown in Table A3.

**Table A3:** Individual concepts performance measures for the Beth, Partner, and Test datasets, employing exact-plus-approximate (hybridized) term matching to UMLS.

Datasets	Categories	Precision (%)	Recall (%)	F1-Score (5)
Beth Medical Center	Problem	73.81%	83.78%	78.48%
	Treatment	72.55%	81.32%	76.68%
	Test	77.14%	44.26%	56.25%
Partners Healthcare	Problem	91.67%	86.52%	89.02%
	Treatment	78.26%	100%	87.80%
	Test	80%	40%	53.33%
i2b2 Test Dataset	Problem	70.15%	77.05%	73.44%
	Treatment	52.81%	78.12%	63.03%
	Test	85.42%	41.79%	56.12%
